# Prognostic Indicators of *EIF1AX*-Mutated Thyroid Tumor Malignancy and Cancer Aggressiveness

**DOI:** 10.3390/cancers14246097

**Published:** 2022-12-11

**Authors:** Saruchi Bandargal, Tanya Chen, Marc Philippe Pusztaszeri, Véronique-Isabelle Forest, Sabrina Daniela da Silva, Richard J. Payne

**Affiliations:** 1 Faculty of Medicine, McGill University, Montreal, QC H3G 2M1, Canada; 2 Department of Otolaryngology–Head and Neck Surgery, University of Toronto, Toronto, ON M5S, Canada; 3 Department of Pathology, McGill University, Jewish General Hospital, Montreal, QC H3T 1E2, Canada; 4 Department of Otolaryngology–Head and Neck Surgery, McGill University, Jewish General Hospital, Montreal, QC H3T 1E2, Canada; 5 Department of Otolaryngology–Head and Neck Surgery, McGill University, Royal Victoria Hospital, Montreal, QC H4A 3J1, Canada

**Keywords:** *EIF1AX* mutation, molecular testing, indeterminate thyroid nodules, thyroid cancer

## Abstract

**Simple Summary:**

Ultrasound-guided fine-needle aspiration (USFNA) biopsy is a widely used first-line diagnostic approach to differentiate between benign and malignant tumors. However, 15–30% of all thyroid nodules investigated by USFNA cytology are indeterminate. To address the elusive clinical management of such nodules, molecular markers for common mutations in thyroid cancer have been researched to serve as prognostic indicators. *EIF1AX* is a rare mutation with no clear prognostic indicators. In this multicenter study of 42 *EIF1AX*-mutated thyroid nodules, we found that 47.6% of nodules were malignant with distinctive risks of malignancy depending on the location of the mutation and the presence of co-mutation(s). An *EIF1AX* A113_splice site mutation in tandem with a *RAS* and/or *TP53* mutation is associated with aggressive malignancies that have an inherent potential to progress toward poorly differentiated thyroid carcinoma.

**Abstract:**

The risk of malignancy (ROM) of *EIF1AX*-mutated thyroid nodules has been theorized to be contingent on the position of the mutation within the gene and the presence of co-existing mutations. However, due to *EIF1AX*’s low mutation frequency, sample sizes currently reported in the literature are too diminutive to appraise the clinical utility of molecular diagnostic testing. The objective of this study was to elucidate prognostic indicators of *EIF1AX*-mutated thyroid tumors and cancer aggressiveness by examining a large cohort of cytologically indeterminate thyroid nodules (CITNs) that underwent molecular testing and subsequent surgical resection. This is a multicenter study involving 764 subtotal and total thyroidectomy patients that underwent preoperative molecular testing at two quaternary care hospitals. A five-year retrospective review was performed on the 42 charts of patients that opted for surgery following a positive *EIF1AX* mutation on ThyroseqV3 results from January 2018 to May 2022. Patient demographics, cytopathology results, molecular testing results, and postoperative histopathology were reviewed. Of the 42 surgically resected nodules that harbored an *EIF1AX* mutation, 16 (38.1%) were benign, six (14.3%) were non-invasive follicular thyroid neoplasms with papillary-like nuclear features (NIFTPs) or well-differentiated thyroid neoplasms of uncertain malignant potential (WDT-UMPs), and 20 (47.6%) were malignant. An isolated *EIF1AX* mutation conferred a ROM of 47.6%, whereas the ROM for nodules with at least one additional molecular alteration was 72.7%. The ROM increased to 100% for nodules with at least one additional molecular alteration and the A113_splice site mutation. Six malignant nodules were aggressive, with five having variegated components of poorly differentiated thyroid carcinoma (PDTC). *EIF1AX*-mutated thyroid nodules are more susceptible to malignancy in the presence of the A113_splice site mutation and when co-mutated with *RAS* and/or *TP53*. This deleterious amalgam is associated with aggressive disease and renders these nodules PDTC. A preoperative molecular test finding of an *EIF1AX* mutation can be a useful tool for thyroid specialists to optimize clinical management.

## 1. Introduction

Over the past three decades, the incidence of thyroid cancer has been increasing worldwide [1]. Heightened surveillance and adoption of diagnostic tools may account for the rising number of early-stage, asymptomatic thyroid cancer diagnoses [2]. As part of routine workup, cytological diagnosis of thyroid nodules by ultrasound-guided fine-needle aspiration (USFNA) biopsy is a cost-effective and highly reliable procedure [3]. USFNA results are useful for stratifying malignancy risk and determining the aptness of surgery. The Bethesda System for Reporting Thyroid Cytopathology is a 6-category classification system created to standardize the interpretation and reporting of thyroid cytology [4]. Management decisions become obscure when the USFNA result demonstrates a Bethesda diagnostic category III or IV, defined as “atypia of undetermined significance or follicular lesion of undetermined significance (AUS/FLUS)” or “follicular neoplasm/suspicious for follicular neoplasm (SFN)”, respectively [3,5]. Molecular testing of thyroid nodules has emerged as an efficacious, ancillary tool for evaluating these cytologically indeterminate thyroid nodules (CITNs) and guiding clinical management [6].

Amongst the most common genetic mutations in thyroid cancer such as *RAS* or *BRAF*, one lesser-known mutation is *E1F1AX* [7]. The Cancer Genome Atlas (TCGA) study was the first landmark study to describe the mutation of *EIF1AX* in thyroid tumors, more specifically in papillary thyroid carcinoma (PTC) [8]. The *EIF1AX* gene is located on chromosome X and codes for a eukaryotic translation initiation factor 1A (eIF1A) [9]. This protein is essential for the recruitment of the ternary complex and for assembling the 43S preinitiation complexes. Hence, together with other translation initiation factors, it is involved in a sophisticated scanning system responsible for accurately locating the correct start codon on eukaryotic mRNA [10]. Deregulation of translation initiation is common in the tumorigenesis of thyroid cancer. Notably, the C-terminal *EIF1AX* A113_splice mutation is the most prevalent in aggressive thyroid cancer [11]. *EIF1AX* A113_splice variants stabilize the preinitiation complexes and induce ATF4, a sensor of cellular stress, enabling a general increase in protein synthesis [11]. Since The Cancer Genome Atlas (TCGA) study, *EIF1AX* mutations have been primarily reported in poorly differentiated thyroid carcinoma (PDTC) and anaplastic thyroid carcinoma (ATC), but also in well-differentiated thyroid carcinoma (WDTC) and benign thyroid nodules, although less frequently [12].

The likelihood of malignancy in an *EIF1AX*-mutated thyroid nodule is nebulous in the literature; its variability has rendered standardized management of such nodules challenging for otolaryngologists. Nonetheless, few studies hypothesize that the risk of malignancy (ROM) may correlate with the presence of co-existing mutations and with the position of the *EIF1AX* mutation within the gene [13,14]. Moreover, histopathological outcomes and the aggression profile of thyroid nodules harboring an *EIF1AX* mutation are poorly understood. The aim of this study is to explicate prognostic indicators of *EIF1AX*-mutated thyroid tumors and cancer aggressiveness by examining the largest cohort to date of CITNs that underwent molecular testing and subsequent surgical resection.

## 2. Materials and Methods

### 2.1. Study Design

This study is a multicenter retrospective chart review involving 764 subtotal and total thyroidectomy patients that previously underwent molecular testing at two quaternary-level hospitals in Montreal, Canada (Jewish General Hospital and Royal Victoria Hospital). Data on patient demographics, preoperative USFNA, molecular testing, and postoperative pathology was collected. Ethics approval was obtained by the Medical-Bioethics Research Ethics Committee (REC) of the Integrated Health and Social Services Network for West-Central Montreal (#MP-05-2022-3268).

### 2.2. Patient Population

The charts of patients ages 18 years or older who underwent preoperative molecular testing using ThyroSeqV3 preceding surgery between January 2018 and May 2022 were screened.

ThyroSeqV3 is a 112-gene, DNA and RNA-based, targeted next-generation sequencing assay that tests for five classes of genetic alterations: (i) point mutations, (ii) indels, (iii) gene fusions (GF), (iv) copy number alterations (CNAs), and (v) gene expression alterations (GEAs). GEA is performed by comparing messenger RNA expression detected in a thyroid USFNA against a panel of 167 genes. Of these 167 genes, 142 are involved in an algorithm that identifies a benign gene expression pattern, and the other 25 genes are involved in filtering out rare neoplasms and assessing for *BRAF V600E* mutations [15].

**Figure 1:**
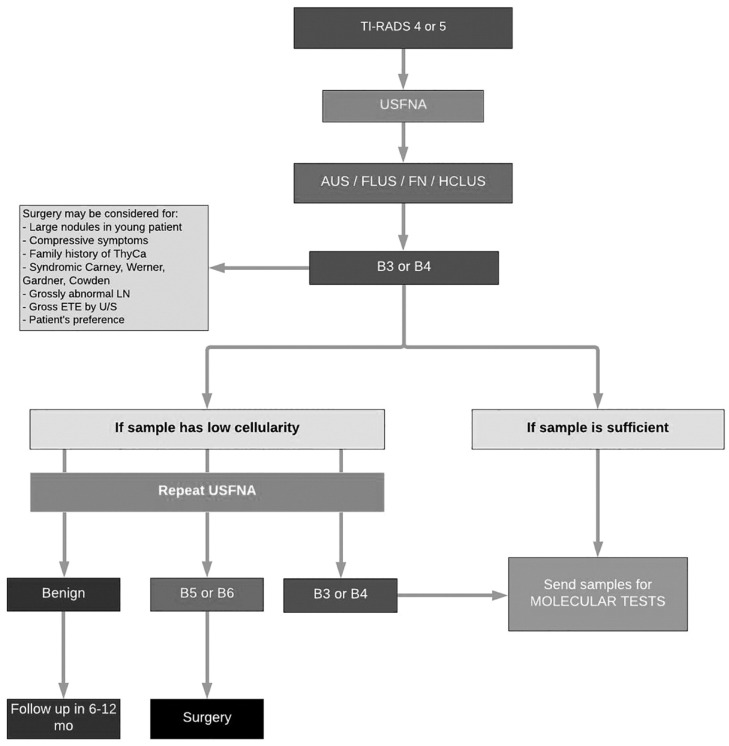
McGill algorithm for workup of indeterminate thyroid nodules. AUS: Atypia of undetermined significance; ETE: Extrathyroidal extension; FLUS: Follicular lesion of undetermined significance; FN: Follicular neoplasm; HCLUS: Hurthle cell lesion of undetermined significance; LN: Lymph node; ThyCa: Thyroid cancer; TI-RADS: Thyroid Imaging Reporting and Data System; U/S: Ultrasound; USFNA: Ultrasound-guided fine-needle aspiration.

Patients underwent molecular testing according to the McGill algorithm for workup of indeterminate thyroid nodules (Figure 1), a novel and interdisciplinary protocol conceived by thyroid cancer specialties at our institution [16]. Patients were eligible for molecular testing if they had a Bethesda III or Bethesda IV nodule. Alternatively, molecular testing was warranted for patients with a Bethesda V nodule if, after consideration of clinical and sonographic features, the test result was expected to alter surgical decision making, accordant with the 2015 American Thyroid Association Management Guidelines [17].

Consent for molecular testing was obtained through a general written consent form used at both hospitals for all surgery, anesthesia, diagnostic, or therapeutic procedures. Only patients who tested positive for an *EIF1AX* mutation were included in the study. All included patients had a thyroidectomy (subtotal or total), a sentinel lymph node biopsy, and a limited central compartment neck dissection.

### 2.3. Tumor Analysis

Two USFNA nodule samples were collected for each patient by a thyroid surgeon. One sample was transported to a commercial laboratory at the University of Pittsburgh Medical Center for ThyroSeqV3 molecular testing. These samples were analyzed for molecular alterations, including genetic mutations. The other sample was sent to the pathology department at the affiliated hospitals for typical cytopathological analysis and a Bethesda score was assigned.

Board-certified head and neck fellowship-trained pathologists reviewed surgical resection specimens for aggressive features. Aggressive features were defined by our pathologists by the presence of one or more of the following: macroscopic extrathyroidal extension (ETE), lymph node metastasis (LNM), poorly differentiated thyroid carcinoma (PDTC), and high-risk histological features (tall cell, columnar cell, hobnail/micropapillary, and diffuse sclerosing).

Three study groups were established based on postoperative pathology diagnosis: benign disease, non-invasive follicular thyroid neoplasm with papillary-like nuclear features (NIFTP) or a well-differentiated thyroid neoplasm of uncertain malignant potential (WDT-UMP), and malignant disease. All thyroid tumors were classified in keeping with the latest WHO 2022 classification of thyroid tumors [18]. Accordingly, PDTC diagnosis is based on the Turin consensus criteria: (i) presence of a solid/trabecular/insular pattern of growth in a tumor diagnosed as malignant based on invasive properties; (ii) absence of conventional nuclear features of papillary carcinoma; (iii) presence of at least one of the following: convoluted nuclei, mitotic count ≥3 per 2 mm^2^, tumor necrosis [18].

### 2.4. Statistical Analysis

Descriptive statistics were performed. For frequency analysis in contingency tables, statistical analyses of associations between variables were performed using a Chi-square test or Fisher’s exact test (with significance set at *p* < 0.05). For continuous variables, the non-parametric Mann–Whitney U tests were used. Statistical analyses were performed using STATA^®^ (STATA Corp., College Station, TX, USA).

## 3. Results

### 3.1. Baseline Characteristics

Of the total 764 patients screened, 42 harbored an *EIF1AX* mutation and were included in our study, resulting in an overall frequency of 5.5%. The mean age was 61 years old, with females being the prominent sex (81%, n = 34). The mean nodule size was 2.2 cm (range <0.1 cm–5.0 cm). Of all nodules, 29 (69%) had a cytologic diagnosis of Bethesda III, 12 (28.6%) of Bethesda IV, and 1 (2.4%) of Bethesda V. The clinical and pathologic features of all 42 patients with full diagnostic characteristics are shown in Table 1.

### 3.2. Tumor Characteristics

**Figure 2:**
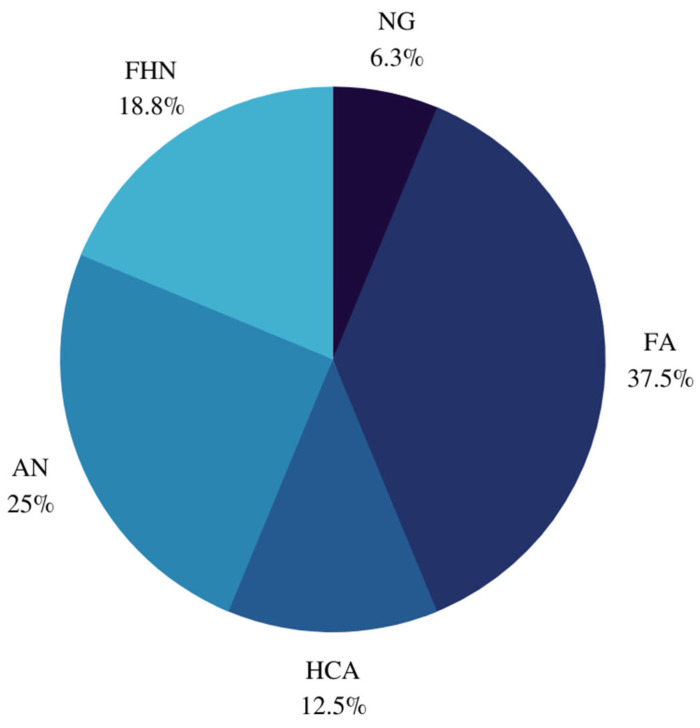
Histopathological diagnoses of benign nodules. NG: Nodular goiter. FA: Follicular adenoma; HCA: Hurthle cell adenoma, AN: Adenomatoid nodule, FHN: Follicular hyperplastic nodule.

**Figure 3:**
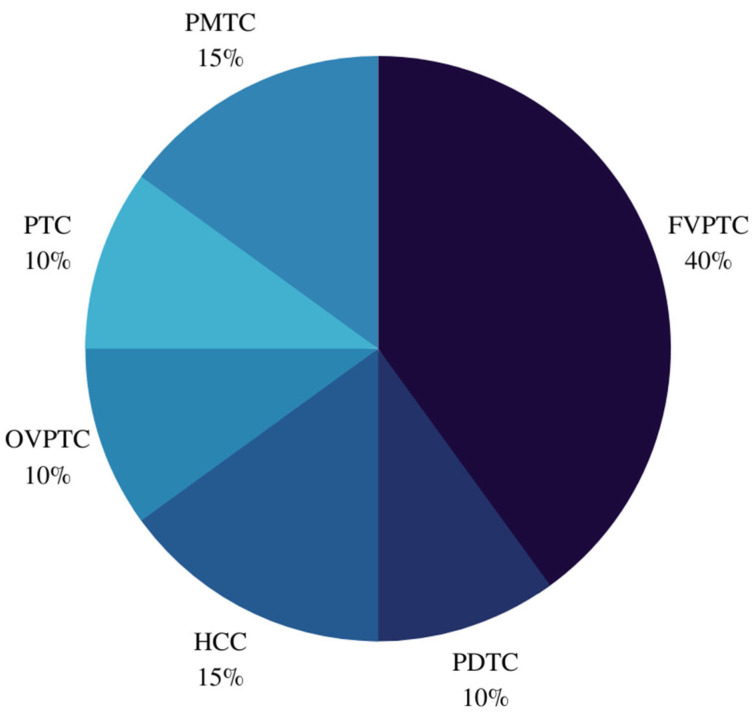
Histopathological diagnoses of malignant nodules. PMTC: Papillary thyroid microcarcinoma; FVPTC: Follicular variant of papillary thyroid carcinoma; PDTC: Poorly differentiated thyroid carcinoma; HCC: Hurthle cell carcinoma; OVPTC: Oncocytic variant of papillary thyroid carcinoma; PTC: Papillary thyroid carcinoma.

Of the 42 *EIF1AX*-mutated nodules, 16 (38.1%) were benign, 6 (14.3%) were NIFTP/WDT-UMP, and 20 (47.6%) were malignant. Of the 15 PTCs, 8 were follicular variants of PTCs (FVPTCs). Classification of FVPTCs revealed two infiltrative FVPTCs and four encapsulated FVPTCs, of which two were aggressive. Histopathological features of these nodules are reported in Table 1. When substratified (Figure 2), eight benign nodules were hyperplastic, of which three follicular nodules had oncocytic metaplasia. Similarly, histopathological diagnoses for malignant nodules are presented in Figure 3.

Eleven of the *EIF1AX*-mutated nodules had at least one additional molecular alteration. Nine had co-existing genetic mutations, including *NRAS*, *HRAS*, *TP53*, *PIK3CA*, *TERT*, and *GNAS.* Four had other additional molecular alterations, three of which had CNAs and one with GEAs. Furthermore, two nodules had co-existing genetic mutations in tandem with CNAs or GEAs. *EIF1AX* mutation alone conferred a 47.6% risk of malignancy (ROM) and 61.9% ROM or NIFTPs/WDT-UMPs, whereas the ROM for nodules with at least one additional molecular alteration was 72.7%. More specifically, the ROM was 100% in nodules with concurrent *NRAS*, *HRAS*, *TP53*+*NRAS*, and *PIK3CA*+*TERT* mutations. However, the ROMs for co-existing *GNAS* mutation and for co-existing *TP53* mutations were 0% and 66.7%, respectively. Furthermore, 17 nodules (40.5%) harbored an A113_splice site mutation at the junction of intron 5 and exon 6. The ROM for this type of *EIF1AX* mutation was 70.6%. The ROM for nodules with at least one additional molecular alteration and the A113_splice site mutation was 100%. The ROMs for A113_splice site-mutated and non-A113_splice site-mutated nodules are summarized in Table 2. Notably, 60% of malignant nodules and 31. Of the benign nodules, 25% possessed the A113_splice site mutation, albeit all NIFTPs/WDT-UMPs were non-A113_splice site *EIF1AX*-mutated.

Of the 20 malignant nodules, 6 demonstrated aggressive features, resulting in an *EIF1AX*-mutated cancer aggressiveness frequency of 30%. Five (83.3%) aggressive malignancies were PDTC, with three having varying components of PDTC alongside other histopathologic diagnoses. Two of the three (66.7%) *EIF1AX+TP53* nodules were malignant and aggressive, both having components of PDTC. Moreover, all aggressive malignancies possessed an A113_splice site mutation, while 50% of them had at least one co-existing genetic mutation, such as CNAs and/or GEAs. Three out of four (75%) nodules with at least one additional molecular alteration and the A113_splice site mutation were aggressive.

The benign group had a mean allele frequency (AF) of 25.4%, the NIFTP/WDT-UMP group had a mean AF of 20.8%, and the malignant group had a mean AF of 25.1%. The mean AF for nodules with the A113_splice site mutation was 28.2%, while that of aggressive malignancies was comparable at 29%. The difference in mean AF between the three study groups was statistically insignificant. Out of the 16 (6.25%) benign nodules, 1 had CNAs, whilst 2 out of the 20 (10%) malignant nodules had CNAs. No NIFTPs/WDT-UMPs had CNAs. One of six (16.7%) aggressive malignancies had CNAs.

## 4. Discussion

Over the past decade, the diagnostic capability of molecular testing has significantly increased due to the emergence of molecular markers designed to accurately rule in or rule out thyroid malignancy. Thus, this tool is quickly emerging as an instrumental tool in the routine workup of CITNs and may eventually become the gold standard for triaging these nodules into those requiring surveillance from those requiring surgery [17,19].

The results of our study demonstrated the high malignant potential of *EIF1AX*-mutated thyroid nodules with a 61.9% ROM or NIFTPs/WDT-UMPs. Most of the tumors were ultimately PTCs, in concurrence with the TCGA study [8]. The majority of PTCs in the TCGA study were FVPTCs, accounting for 83.3% of PTCs [8]. Likewise, the most prevalent variant of PTC in our study was the follicular variant. Karunamurthy et al. found that in their 2016 cohort of surgically resected specimens, all three of their *EIF1AX*-mutated PTCs were encapsulated FVPTCs, and appositely suggested that this low-risk diagnosis was a typical phenotype of *EIF1AX*-mutated PTC (*RAS*-like tumors) [13]. By contrast, our study demonstrated the presence of more aggressive PTC variants such as infiltrative variants (*BRAF*-like tumors) that often harbor nodal metastases, as well as aggressive encapsulated FVPTCs [20]. Therefore, when considering clinical management for *EIF1AX*-mutated thyroid nodules, the mutation’s aggressive potential should be taken into consideration.

Karunamurthy et al.’s study was the first to acknowledge the presence of *EIF1AX* mutations in benign thyroid nodules [13]. Six benign nodules were follicular adenomas (FAs), while no follicular thyroid carcinomas (FTCs) were detected in our analysis. Strikingly, eight PTCs were FVPTCs. This similitude of prevalence may posit, in accord with the aforementioned study, that under the impact of *EIF1AX* mutation, FAs have a proclivity to progress to FVPTC, instead of FTC [13]. We had two hurthle cell adenomas and three follicular nodules with oncocytic metaplasia. Oncocytic change is seen in follicular nodular disease and adenomas that have mitochondrial DNA alterations and chromosomal gains/losses that scarcely differ between benign and malignant nodules [21]. Furthermore, there has been dissent on the diagnosis of oncocytic follicular adenomas since some authors have found that these benign lesions subsequently behaved in a malignant fashion, sometimes developing metastases [22]. Hence, these nodules, along with FAs, may have the propensity to progress to NIFTPs/WDT-UMPs and malignant tumors with time.

*EIF1AX* mutation appears to frequently coincide with other mutations, particularly *RAS*. In our study, the co-occurrence of *NRAS*, as well as *HRAS* elevated the ROM two-fold and yielded two aggressive malignancies. The poor prognostic combination of *EIF1AX*+*RAS* was first observed in the TCGA study, and several studies, including ours, have since corroborated its aggressiveness [8,11,12,23]. As initially introduced by Krishnamoorthy et al., we hypothesize that *EIF1AX* mutations may require their *RAS* counterpart to fully progress into apparent malignancy [11]. Concurrent *TP53* conferred a higher risk of aggressiveness which has not been previously reported in the literature. Table 3 summarizes previously published studies on *EIF1AX*-mutated thyroid nodules that underwent molecular testing and subsequent surgical resection. Furthermore, the *EIF1AX* A113_splice mutation was associated with a higher ROM with association to AF. While there seems to exist a correlation between the A113_splice mutation and aggressiveness, further studies are needed to attest to causation. Co-existing molecular alterations conferred a higher ROM, which has been described in previous studies due to the synergistic effect of co-mutations that propel tumorigenesis [11]. All three nodules bearing an *EIF1AX-A113_splice* mutation in conjunction with *RAS* and/or *TP53* were aggressive malignancies with components of PDTC. Based on these findings, nodules with concurrent *RAS* and/or *TP53* mutations and the A113_splice site mutation should undergo surgical intervention as they may have a high lifetime ROM, notwithstanding being benign on initial testing. This mutational profile may mark the beginning of a stepwise progression of *EIF1AX+RAS/TP53*-driven thyroid cancer.

A limitation to our study is the selection bias caused by the high cost of molecular testing, which is available only as an out-of-pocket expense, often a prerogative of those with a higher socioeconomic status. Thus, not all patients with CITNs opted for molecular testing, perhaps underrepresenting the number of *EIF1AX*-mutated thyroid nodules. Another limitation to our study design is the strict selection criteria compared to studies that included all Bethesda nodules. This was done in order to reflect more realistically the challenge of clinical management for CITNs. This study was devoid of ascertaining recurrence rates, additional treatment options to optimize surgical intervention, or prospective progression of benign nodules as we did not assess follow-up information for patients who underwent surgery and those who opted for conservative management. Further studies should include benign *EIF1AX*-mutated nodules to test their potential for progression to NIFTPs/WDT-UMPs and/or malignant nodules in the absence of surgery. Lastly, as our hospitals are quaternary-level hospitals, the referral pattern might lean towards a biased increase in patients with aggressive malignancies.

## 5. Conclusions

This study demonstrates the viability of molecular diagnostic testing in identifying prognostic factors of *EIF1AX*-mutated CITNs, thus preventing unnecessary diagnostic surgery and improving clinical management. We found that select benign nodules might inherently be predisposed to acquiring NIFTP/WDT-UMP or malignant features with time. A molecular test finding of the A113_splice site with concurrent *RAS* and/or *TP53* may warrant surgical intervention as such nodules have a relatively high propensity to be malignant. This pernicious combination is also associated with aggressive disease, rendering these nodules PDTC. Molecular profiles might help predict ROM and risk of aggressiveness when confronted with an *EIF1AX*-mutated CITN.

## Figures and Tables

**Table 1:** Clinicopathological features of *EIF1AX*-mutated cytologically indeterminate thyroid nodules by study group.

Case	Sex	Age (Year)	Cytologic Diagnosis (Bethesda Score)	Histopathologic Diagnosis	Nodule Size (cm)	Co-Existing Mutation(s)	Type of *EIF1AX* Mutation	*EIF1AX* Mutation AF (%)	Co-Existing Mutation(s) AF (%)	Aggressive Feature
1	F	60	4	FA	0.9	-	A113_splice site	15	-	-
2	F	69	3	NG	1.8	-	R13L	38	-	-
3	F	55	3	AN	0.8	-	A113_splice site	8	-	-
4	F	55	3	FA	1.4	-	G9V	28	-	-
5	F	62	3	AN	1.5	*TP53*	G15D	2	9	-
6	F	63	3	FA	1.5	-	G9R	28	-	-
7	F	58	3	FHN	1	-	A113_splice site	36	-	-
8	M	66	3	AN	1.8	-	A113_splice site	59	-	-
9	F	70	3	FA	2.6	CNAs	G9R	27	-	-
10	F	46	3	FA	3	-	N11_E20dup	14	-	-
11	F	82	3	FHN	-	-	G6V	12	-	-
12	M	43	3	FA	5	-	A113_splice site	14	-	-
13	F	68	3	HCA	1	-	G124L	14	-	-
14	F	78	3	HCA	3.4	-	K10_N11insKPSGKGGK	10	-	-
15	F	85	4	FHN	-	-	G9R	40	-	-
16	M	52	3	AN	4	-	G8E	62	-	-
17	F	72	3	FVPTC	1.3	*NRAS*	R13P	11	10	-
18	F	75	4	PDTC	2.5	-	A113_splice site	12	-	PDTC
19	F	68	3	HCC	3	*TP53*	A113_splice site	30	45	40% component PDTC
20	F	88	3	IFVPTC	2.8	-	G9V	22	-	-
21	F	51	4	IFVPTC	1.8	*NRAS*	G9D	26	23	-
22	F	77	4	OVPTC	0.6	-	A113_splice site	18	-	-
23	F	75	4	PDTC	3.5	-	A113_splice site	19	-	PDTC
24	F	44	4	EFVPTC	3.9	-	A113_splice site	24	-	Positive SLNB
25	F	77	3	HCC	4	*PIK3CA*, *TERT*	N17_K23dup	11	16, 16	-
26	F	45	3	PTC	0.55	-	A113_splice site	6	-	-
27	M	60	4	HCC	-	*TP53*, *NRAS*, CNAs	A113_splice site	57	40, 7	30% component PDTC
28	M	74	4	EFVPTC	5	*HRAS*, GEAs	A113_splice site	32	19	10% component PDTC
29	F	58	4	PTC	2	-	A113_splice site	37	-	-
30	F	44	3	PMTC	0.3	-	G8R	17	-	-
31	M	33	3	EFVPTC	5	*HRAS*	A113_splice site	61	29	-
32	F	77	3	OVPTC	3.5	-	A113_splice site	20	-	-
33	M	55	3	PMTC	0.3	-	G5V	23	-	-
34	F	49	3	EFVPTC	2	-	A113_splice site	32	-	-
35	F	64	4	FVPTC	-	CNAs	G8V	29	-	-
36	F	66	3	PMTC	0.1	-	R13P	15	-	-
37	M	58	3	WDT-UMP	3.2	-	K16E	29	-	-
38	F	48	3	NIFTP	1.3	-	G9D	15	-	-
39	F	46	3	WDT-UMP	2.5	-	G9D	10	-	-
40	F	26	4	WDT-UMP	2.1	*GNAS*	G15D	11	10	-
41	F	66	5	NIFTP	2	-	R13H	29	-	-
42	F	68	3	NIFTP	0.7		G9V	31	-	-

Cases 1–16: Benign nodules; Cases 17–36: Malignant nodules; Cases 37–42: NIFTPs/WDT-UMPs. FA: Follicular adenoma; NG: Nodular goiter; AN: Adenomatoid nodule; FHN: Follicular hyperplastic nodule; HCA: Hurthle cell adenoma; FVPTC: Follicular variant of papillary thyroid carcinoma; PDTC: Poorly differentiated thyroid carcinoma; HCC: Hurthle cell carcinoma; IFVPTC: Infiltrative FVPTC; OVPTC: Oncocytic variant of papillary thyroid carcinoma; EFVPTC: Encapsulated FVPTC; microMTC: Medullary thyroid microcarcinoma; PTC: Papillary thyroid carcinoma; PMTC: Papillary thyroid microcarcinoma; WDT-UMP: Well-differentiated thyroid neoplasms of uncertain malignant potential; NIFTP: Non-invasive follicular thyroid neoplasm with papillary-like nuclear features; SLNB: Sentinel lymph node biopsy.

**Table 2:** ROMs for non-A113_spice site and A113_spilce site mutations.

Type of *EIF1AX* Mutation	ROM (%)	ROM or NIFTPs/WDT-UMPs (%)	ROM for Nodules with ≥1 Additional Molecular Alteration (%)	ROM for Nodules with Concurrent *RAS* (%)	ROM for Nodules with *TP53* (%)
All	47.6	61.9	72.7	100	66.7
non-A113_splice site	36	56	–	100	0
A113_splice site	70.6	70.6	100	100	100

**Table 3:** Comparison of key results in present and previously published studies.

Study	Sample Size n (%)	Mutation Frequency (%)	Benign Disease n (%)	NIFTPs/WDT-UMPs n (%)	Malignancy n (%)	A113_Splice Site Mutation (%)	Nodules with ≥1 Additional Molecular Alterations (%)	Co-Existing Genetic Mutations Present	Nodules with Concurrent *RAS * (%)	Nodules with Concurrent *TP53* (%)
**Current study** Sensitivity Specificity PPV	42 (5.5)	5.5	16 (38.1)	6 (14.3)	20 (47.6)	40.5 57.2 76.2 70.6	26.2 53.4 81.8 77.8	*TP53*, *NRAS*, *PIK3CA*, *TERT*, *HRAS*, *GNAS*	11.9 23.8 100 100	7.14 9.5 95.2 66.7
**Elsherbini et al., 2022 [24]** PPV	31 (5)	5	17 (55)	2 (6.5)	12 (38.7)	48.4 53	22.6 85.7	*NRAS*, *HRAS*, *TP53*, *TERT*, *PIK3CA*	12.9 100	9.7 66.7
**Karslioglu et al., 2022 [25]** PPV	31 (5)	-	17 (55)	2 (6)	12 (39)	45 33.3	48 80	-	-	-
**Gargano et al., 2021 [26]** * PPV	26 (4.5)	4.5	6 (23)	3 (11.5)	17 (65.4)	65.4 85	57.7 93	*KRAS*, *NRAS*, *TERT*, *HRAS*, *TP53*, *YWHAG-BRAF*	46.2 91.7	7.7 100
**Karunamurthy et al., 2016 [13]** * PPV	11 (4.2)	4.2	7 (63.6)	0	4 (36.4)	54.5 83.3	27.3 100	*NRAS*, *TP53*, *TERT*	27.3 100	9.1 100

* Studies comprised surgically resected and non-resected *EIF1AX*-mutated nodules; however, only those with a postoperative histopathology report were included. Sensitivity and specificity analyses from previously published studies were not publicly available. PPV: positive predictive value; n: total number of cases.

## Data Availability

The data presented in this study are available on request from the corresponding author. The data are not publicly available due to the ethics approval agreement.
